# Diuretin and Diuretics—Practical

**Published:** 1894-03-03

**Authors:** Benjamin Ward Richardson


### DIURETIN AND DIURETICS.—PRACTICAL.

By Sir Benjamin Ward Richardson, M.D., F.R.S.

I proceed to consider the subject of my last paper on the practical side, dealing with the value of the substance called diuretin in the treatment of disease. My first observations were made, if I may so say, casually, that is, I began to prescribe diuretin in small, increasing, and large doses, in cases where a diuretic was supposed to be required, without special reference to cause. The first few administrations gave colour to the idea that diuretin possesses the general powers claimed for it; but I soon found great variability in regard to its action, and was led to the conclusion to which Dr. H. A. Hare had arrived, that the greatest care must be taken in observation, in order that other agencies affecting the excretion of urine should not interfere with the correctness of the observer. It also seemed to me that in some instances symptoms followed the administration of the drug which were of a general kind, such as headache, depression, a little fever, somnolency, or slight delirium, symptoms which were not actually dangerous or alarming in degree, but which were inconvenient, and which called for cessation of the administration, at least temporarily.

I never witnessed, however, the symptoms of vomiting and diarrhœa which some have observed as after effects, and I considered that there was no direct evidence of cumulative action such as is observed from digitalis. Altogether, I looked upon diuretin as being a diuretic under some circumstances, but as one rather uncertain in its effects. In order, therefore, to watch those effects in a more systematic way, I noted them carefully in one or two particular cases where other diuretics had been prescribed.

A man, 62 years of age, came under my care, in hospital, on July 9th, 1892. He weighed 210 lbs., and stood 6 ft. 2 in. in height. He had been a night watchman, and had remained at his occupation almost up to the date of his admission. He complained of great pain in his right side, and on careful examination was found to have a considerable effusion in the right pleural cavity, with probably some ascites. It was observed that he suffered from shortness of breath, especially at night, and a day or two after admission 88 fluid ounces of a clear serous fluid was removed from the chest by aspiration. The removal gave immediate relief, but the fluid soon re-accumulated, and three weeks later 124 ounces more were drawn off. Fluid also was collecting in the abdomen; and, to be brief, between August 17th, 1892, and May 20th, 1893, when the patient was discharged to go to a home, he had been tapped seven times in the thorax, fourteen times in the abdomen, and twice—by Southey's tubes—in the lower limbs, by which operations 632 ounces were drawn off from the chest, 3,089 ounces from the abdomen, and 272 from the lower limbs, in all, 24 gallons 3 quarts 1 pint and 13 fluid ounces. He was greatly relieved each time by the removal of fluid, and, as his heart was natural, our diagnosis was, chronic nephritis, with cirrhosis or chronic contraction of the kidneys. There was never any febrile state, but, on the contrary, a slightly subnormal temperature as a rule. He took his food well, and up to his discharge was in good spirits.

As may be expected, we did our best in this case to promote action of the kidney. There was no regular appearance of albumen in the urine, but the amount of urine secreted was at all times diminished, often to an extreme degree, so that some days 14 ounces was the most passed, during which the reaccumulation of fluid in the abdomen and chest was rapidly increased, as if these cavities became supplementary receptacles for the water that ought to have passed off through its natural channel. In the course of general treatment the patient was of course judiciously limited, as to the amount of watery fluids which he imbibed, and I think I may say every reasonable form of diuretic was administered to him, without giving him any relief, and without increasing in the least degree the quantity of the excretion. The case thus became one singularly good for testing the value of diuretin as a diuretic, and on March 17th, 1893, diuretin was administered in 5-grain doses, which were continued for three days without striking effect. The dose was then gradually increased to 10 grains with such good action that after administration three times in the 24 hours, 75 ounces of urine were discharged. Free diuresis was maintained for several days, when it gradually failed, with appearance of symptoms which forbade the continuance of the drug. There was headache, considerable depression, undue somnolency, and a rise of temperature, symptoms which passed away within twenty-four hours on the discontinuance of the remedy. After the lapse of a few days, the amount of urine having become greatly diminished, I represcribed the diuretin in 10-grain doses without any definite action, but with recurrence of the objectionable general symptoms.

In this instance diuretin failed to sustain free diuretic action, and induced systemic symptoms similar to those which follow the administration of soda salicylate and sometimes salicylic acid, but without the deafness or noises in the head which occasionally follow the action of these drugs when they are caried to a toxic degree.

The action of the diuretin in the case given was, I should assume, on the renal epithelium, leading in the end to obstruction with an after effect on the nervous system, produced either by the salt itself or by retention of urea, or a combination of both effects. That the action was not cumulative is shown by the fact that the symptoms quickly ceased when the substance causing them was withdrawn.

### DISEASE-PROOF CLOTHING.

The description comes to us from across the Atlantic of a certain "disease proof" suit of clothes to be found in the Patent Office at Washington. The suit is intended to be worn by an operating surgeon. It is a complete suit of rubber armour, resembling, in fact, the dress of an ordinary diver. This is constructed on air-tight principles, and therefore no disease germs can enter. A small pair of bellows are to be found beneath each foot, which, being compressed by the action of walking, blows fresh air in an ingenious manner through the armour. This air enters and is filtered through a germ-proof diaphragm under each of the feet, passing upward and out through a diaphragm which is placed at the top of the head. The description further adds that the operator has protection afforded to his visual organs through two glass eye pieces.

